# Causes of pre and post-donation deferrals among blood donors, at Kwale Satellite Blood Transfusion Center, Kwale County, Kenya, 2018–2022

**DOI:** 10.1186/s12889-024-19535-1

**Published:** 2024-08-13

**Authors:** Gibson Waweru Nyamu, Matano Ali Shee, Polly Kiende, Benson Muithya Muthiani, Rocky Jumapili Nakazea, Narcis Mwakidedela Mwasowa, Frendrick Odhiambo, Jacintah Mueni King’oo, Ronald Nyarambe Wigina

**Affiliations:** 1 School of Applied and Health Sciences, Department of Medical Sciences, Technical University of Mombasa, P.O Box 90420,–80100, Mombasa, Kenya; 2 Ministry of Health, P.O. Box: 30016–00100, Nairobi, Kenya

**Keywords:** Blood donation deferrals, Transfusion transmissible infections, HIV, Hepatitis B, Hepatitis C, Blood donors, Syphilis

## Abstract

**Background:**

Both pre-donation and post-donation deferrals pose challenges to blood safety and availability. This study delved into the deferral rates before donations and their underlying reasons, as, transfusion transmissible infections (TTIs) leading to post-donation deferrals among potential blood donors at the Kwale Satellite Blood Transfusion Centre (KSBTC) in Kenya.

**Methods:**

We performed a retrospective electronic record review of pre- and post-donation deferrals among blood donors at KSBTC, 2018–2022. The pre-donations deferral rate and reasons for deferral were analyzed. Accepted donations were analyzed to determine the prevalence of HIV, hepatitis B (HBV), hepatitis C (HCV), and syphilis. Descriptive statistics were calculated and both crude odds ratio (COR) and adjusted odds ratio (AOR), and their 95% confidence intervals (CI) were calculated. Variables with *p* < 0.05 were considered statistically significant.

**Results:**

A review was conducted on 12,633 blood donation records. Among these, individuals 2,729/12,633 (21.60%) were deferred from donating with the primary reason being low hemoglobin levels, constituting 51.86% of deferrals. Around 773/9,904 (7.80%) of blood units, were discarded due to at least one TTI. Among these, HBV accounted for 4.73%, HIV for 2.01%, HCV for 1.21%, and Syphilis for 0.59% of cases. The adjusted odds ratio for male donors were, (aOR = 1.3, 95% CI 1.01–1.57), donors with none or primary education level (aOR = 1.4 95% CI 1.11–1.68), first-timer donors (aOR = 1.2, 95% CI 1.01–1.44), and static strategy for blood collection (aOR = 1.4, 95%CI 1.12–1.63) were independently potentially associated with testing positive for at least one TTI.

**Conclusion:**

The study indicates that TTIs continue to pose a risk to the safety of Kenya’s bloodstock, with a notable prevalence of HBV infections. Male donors, individuals with limited education, first-time donors, and utilizing a fixed strategy for blood collection were identified as potential risk factors independently associated with TTIs.

**Supplementary Information:**

The online version contains supplementary material available at 10.1186/s12889-024-19535-1.

## Background

Ensuring the safety of blood and its components is crucial, especially since recipients often undergo transfusions during periods of compromised health. Ethics and regulations require blood donation services to minimize health risks for both blood donors and blood recipients. The most susceptible link in the chains of blood and blood components transfusion is donor availability. When donors do not meet the eligibility criteria for donating blood at the blood collection site, a deferral occurs to protect both the donor’s health and that of the recipient. Donor deferrals can be either permanent or temporary. Permanent deferral indicates that prospective donors have enduring health conditions that render them ineligible to donate, such as the risk of transmitting transfusion-transmissible infections (TTIs). On the other hand, temporary deferrals occur when prospective donors have reversible factors, like low blood hemoglobin levels or a short time since their last donation [1].

Transfusion centers have reported pre-donation deferral rates to vary between countries reported; in Tanzania was 12.70% [2], in Cameroun was 13.60% [3], in Nigeria was 8.89% [4], and in India was 8.87% [5], and the causes of pre-donation deferrals include, low hemoglobin levels, hypertension, hypotension, underweight, behavioral risks, low or high pulse rate, being on medication, age-related reasons, among others [1–5].

Post-donation deferrals are primarily attributed to TTIs. Various studies in Kenya have shown a seroprevalence of TTIs ranging from 3.3 to 14.1% [6–9]. In Kenya, the prevalence of Hepatitis B Virus (HBV) infections among blood donors varies from 1.2 to 8.8% [6–10]. Human Immunodeficiency Virus (HIV) infections at blood donation sites range from 0.9 to 5% [6, 7, 9]. The positivity rate for Hepatitis C Virus (HCV) infections in various regional blood donation sites in Kenya spans from 0.5 to 3.2% [6, 7, 9, 11]. Syphilis infections have been reported at rates between 0.6% and 3% [6–10].

Concerning factors associated with TTIs, in Kenya, the study reported no associations between the frequency of TTIs for male (1.9%) versus female (1.5%) donors *p* = 0.470 [8]. Additionally, another study found that female voluntary donors had a 40% higher likelihood of testing positive for HBV infections compared to male donors (*p* = 0.29), but this difference did not reach statistical significant [10]. In Kenya, the study reported no associations between the frequency of TTIs for first-time (3.1%) versus repeat (03%) donors *p* = 0.156 [8], while another study revealed that first-time blood donors had a 50% high chance of testing positive for HBV infections compared to repeat donors (cPOR = 1.5, 95% CI 0.61–3.57, *p*-value 0.38) but were not statistically significant [10]. The prevalence of TTI markers was significantly higher in FRDs compared to VNRDs for HBV (3.6% versus 1.9%, *p* < 0.000), HCV (1.0% versus 0.7%, *p* < 0.006), HIV (0.6% versus 0.3%, *p* < 0.000), and another HIV marker (1.7% versus 0.5%, *p* < 0.000) [12].

According to the World Health Organization, deferral rates have been shown to vary in different setups from less than 1% to over 37%, with a median of 12% [13]. Pre-donation deferrals for whatever reason represent loss of time and effort for both potential donors and personnel working in blood banks.

Kenya has in place a guideline on donor selection and deferral criteria [14]. Donors may be deferred due to various factors such as age, TTIs, social behaviors, travel to infectious countries, medical procedures, medication intake, and pregnancy [14].

In Kenya, all these factors for pre-donation deferrals exist among populations, though their prevalence among the potential donors remains unexplored despite elaborate pre-qualification processes for blood donation that entail; the blood donor must be aged between 16 and 65 years, weighing 50 kg or more, hemoglobin level above or equal to 12.5 g/dl, consent to donate, among others [14].

There are 6 regional blood transfusion centers and 43 satellites in Kenya [14]. According to the WHO, a country needs to donate 1% of its total population’s blood to be self-reliant. For Kenya, this means approximately 500,000 units should be donated annually to meet the blood demand [14, 15]. The population of Kwale County is estimated to be one million, which creates an annual blood demand of approximately 9,000 units based on the 2019 census [16]. For instance, in 2019 and 2020, the national annual blood collection was 136,305 units and 100,108 units, respectively. These amounts fulfilled only 29% and 21.2% of the country’s yearly blood requirements [14]. In 2023, the Kwale Satellite Blood Transfusion Centre (KSBTC), managed to collect a total of 3,484 blood units, accounting for 38.71% of the required amount, leaving 61.29% of the demand unmet (unpublished data). Despite the shortfall in meeting the annual blood collection targets both nationally and in Kwale County, there are additional challenges such as TTIs, blood contamination from air in the bags, clots, and other issues, as well as blood expiring post-collection. These factors threaten blood availability and safety, particularly in low and middle-income countries like Kenya. Blood donors willingly endure the time, energy, and discomfort (needle injection) to help those in need. Investigating the causes and factors leading to donor deferrals is crucial for developing strategies to retain motivated but deferred donors. While several studies in Kenya have focused on specific TTIs among blood donors [6–10], no research has examined the rates and reasons for pre- and post-donation deferrals in transfusion centers and satellites across Kenya. This pioneering study in our region aims to provide a comprehensive analysis of the rate of deferral of blood donors for both pre-and post-donation deferrals, the causes of deferral, and factors associated with the TTIs at the KSBTC. The findings will improve our understanding of the level of adherence to the guidelines on the stipulated criteria for improving the selection of blood donors. The study will also inform more effective planning and policy-making for the development of rational donor recruitment strategies with more substantial criteria, without affecting blood safety and reducing the need to discard donated blood due to TTIs.

## Methods

### Study area

**Fig. 1:**
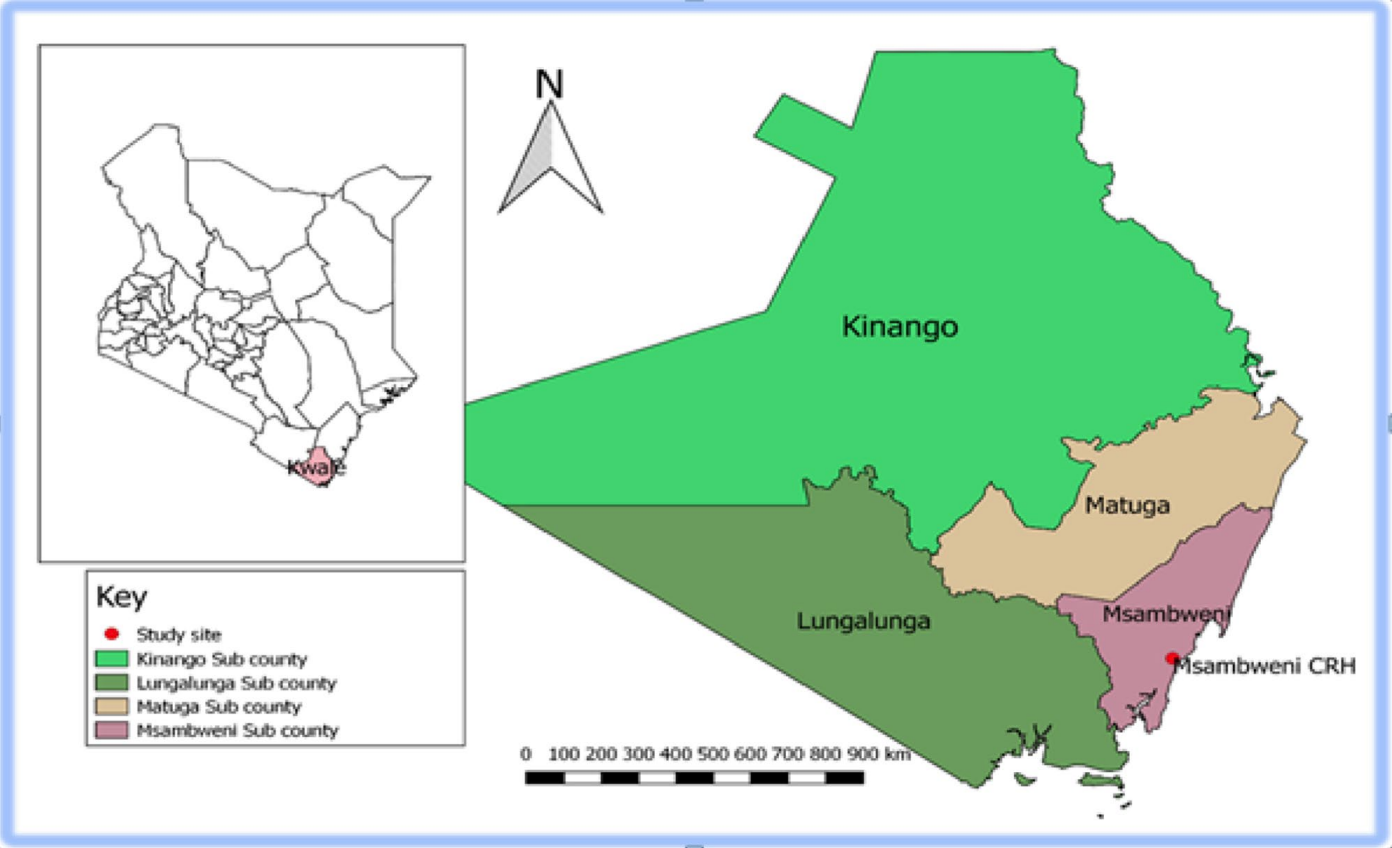
Sampled health facility. (Map developed by the Author using QGIS Version 2.18.10) with geographical data obtained from https://africaopendata.org/dataset/kenya-counties-shape file and data on geo-coordinates and categories of health facilities

This study was carried out among both voluntary and family/relatives blood donors at the Kwale Satellite Blood Transfusion Center (KSBTC), situated within the premises of the Msambweni County Referral Hospital (MCRH) (Fig. 1). The KSBTC utilizes two approaches for blood collection. The primary method involves a mobile strategy targeting VNRDs. Trained medical staff arranges appointments with leaders of various institutions such as schools, colleges, and churches through written letters, emails, and phone calls. They explain the purpose and objectives of organizing blood donation drives in these secondary institutions. Following permission, prospective donors receive a pre-donation health briefing covering the significance of blood donation, donor criteria, and deferral conditions. Prospective donors undergo a national blood transfusion questionnaire for risk assessment. Those meeting the donor selection criteria outlined in national guidelines are allowed to donate blood after consenting to testing for several TTIs including HBV, HIV, HCV, and syphilis. The collected blood is then transported to the KSBTC for TTI screening. The second approach is static, where walk-in donors either come for blood replacements for their acquaintances or, to a lesser extent, voluntarily donate blood at the satellite center. Kwale County has five public and three private hospitals, which act as blood transfusing facilities, and the mandate of KSBTC is to provide the blood and blood components to these facilities and beyond.

### Study design and population

This study was a retrospective using secondary analysis of the archived data from electronically accepted and pre-deferred donor clinical forms among both voluntary and family/relative blood donors, from 2018 to 2022 at KSBTC. The study included records of donors who met the Kenya National Standards for Blood Transfusion Services (KNSBTS) donation [14] requirements consequently, records lacking reasons for deferral were omitted from the analysis.

### Definition of terms

**First-time donors** are donors who attempted to donate blood for the first time in their lifetime.

**Repeat donors** are donors who have donated at least once previously.

**Voluntary Non-Remunerated Donors** are donors who give whole blood, voluntarily without receiving payment in the form of money or a substitute for money, but only for a self-driven sense of altruism or community responsibility. These types of donors were either walk-in to the satellite facility or during outdoor or mobile blood donation drives.

#### Family replacement donors

A friend or family member of the recipient who donates blood to replace the stored blood used for transfusing a recipient, in an attempt to ensure a consistent supply of blood in the satellite blood facility. These types of donations are usually compulsory.

### Data source

This study reviewed electronic clinical donor/deferral forms at KSBTC from 2018 to 2022. The KSBTC maintains donor clinical questionnaire forms and electronic records containing details of accepted and deferred donors.

The electronic spreadsheets have the following variables; age, sex, religion, marital status, education level, occupation, blood group, type of donor, donation strategy, frequency of donation, TTI makers, and reason/s for deferral in case of pre-donation deferral.

### Screening of prospective blood donors and laboratory procedures

The conditions that are considered for deferrals in Kenya include, anyone below 16 years of age, Unexplained recent weight loss of more than 10% of body weight, Less than 56 days after whole blood donation, abnormal blood pressure or heart rate, recent Aspirin use, healthy person but under influence of alcohol at the time of donation, major organ disease (e.g. Heart, liver, lungs) cancer, or abnormal bleeding tendency unless determined eligible by the medical director, recent dental Extraction/ Surgery, pregnancy, presence of TTIs, recent immunization or vaccinations and receipt of blood/ blood components within 1 year.

Prospective donors undergo the national blood transfusion risk assessment through a questionnaire, and those meeting the blood donor selection criteria outlined by Kenya Tissue and Transplant Authority (KTTA) guidelines are permitted to donate. Before donation, they are informed about the testing of various TTIs, including HBV, HIV, HCV, and syphilis, after which they sign, consent form. The collected blood is then packed in a cooler box and sent to the KSBTC for TTI testing.

Blood samples from each donor are placed in sterile serum collection bottles and centrifuged at 1,500 revolutions per minute for 15 min at KSBTC laboratory.

The serum is separated, transferred into sterile cryovials, and stored at 2–8 °C. It undergoes routine testing for HBV, HIV, HCV, and syphilis. The serological tests for anti-HBV, anti-HIV, and anti-HCV are performed using standard enzyme-linked immunosorbent assays (ELISAs), specifically the fourth-generation Murex anti-HBsAg, anti-HIV, and anti-HCV version 4 (manufactured by Diasorin S.p.A UK Branch). Tests with optical density values above the cut-off, according to the manufacturer’s instructions, are considered reactive for anti-HBsAg, anti-HIV, and anti-HCV. Quality controls are applied to each run, including three control wells for both positive and negative results. Known positive and negative samples are also processed alongside the donor samples to ensure accuracy.

For syphilis screening, the presence of antibodies to *Treponema pallidum* is determined using the Rapid Plasma Reagin test (RPR) from Omega Diagnostics Ltd. (Omega, Hill Foots B/V, Alva FK 125 DQ, Scotland, U. K), and results are notified to the clients.

### Data management and analysis

The spreadsheets containing the relevant variables were extracted from the blood donor surveillance system and underwent a thorough data-cleaning process. This was followed by a review for accuracy and respective coding of the variables. The Excel sheets were uploaded and analysis was done using the Epi-info 7 statistical package provided by the Centers for Disease Control and Prevention (CDC) in Atlanta, USA.

Frequencies and proportions were calculated for categorical variables. Means and standard deviations (SD) of continuous symmetrical variables, medians, and interquartile ranges (IQR) for the continuous asymmetrical variables were calculated. Independent variables (age, sex, religion, marital status, education level, occupation, blood group, donation strategy, and frequency of donation) were compared with the dependent variable (donors with at least one TTI) of the prospective donor participants. Both crude odds ratio (COR) and adjusted odds ratio (AOR), and their 95% confidence intervals (CI) were calculated. Variables with *p* < 0.05 were considered statistically significant. Variables with *p*-values ≤ 0.20 were included in a logistic regression model using a backward stepwise elimination method to identify independently associated factors with TTIs.

### Ethical consideration

This study was approved by the Technical University of Mombasa Scientific and Ethics Review Committee (TUM ERC BSC/116/2023). Further permission to conduct the study was granted by the Kwale County, Department of Health and the director of KSBTC.

The study participant’s identity was fully anonymized during data acquisition and analysis to maintain confidentiality. The source documents were stored in a lock and key cupboard and the electronic version was stored in password-protected computers.

## Results

**Fig. 2:**
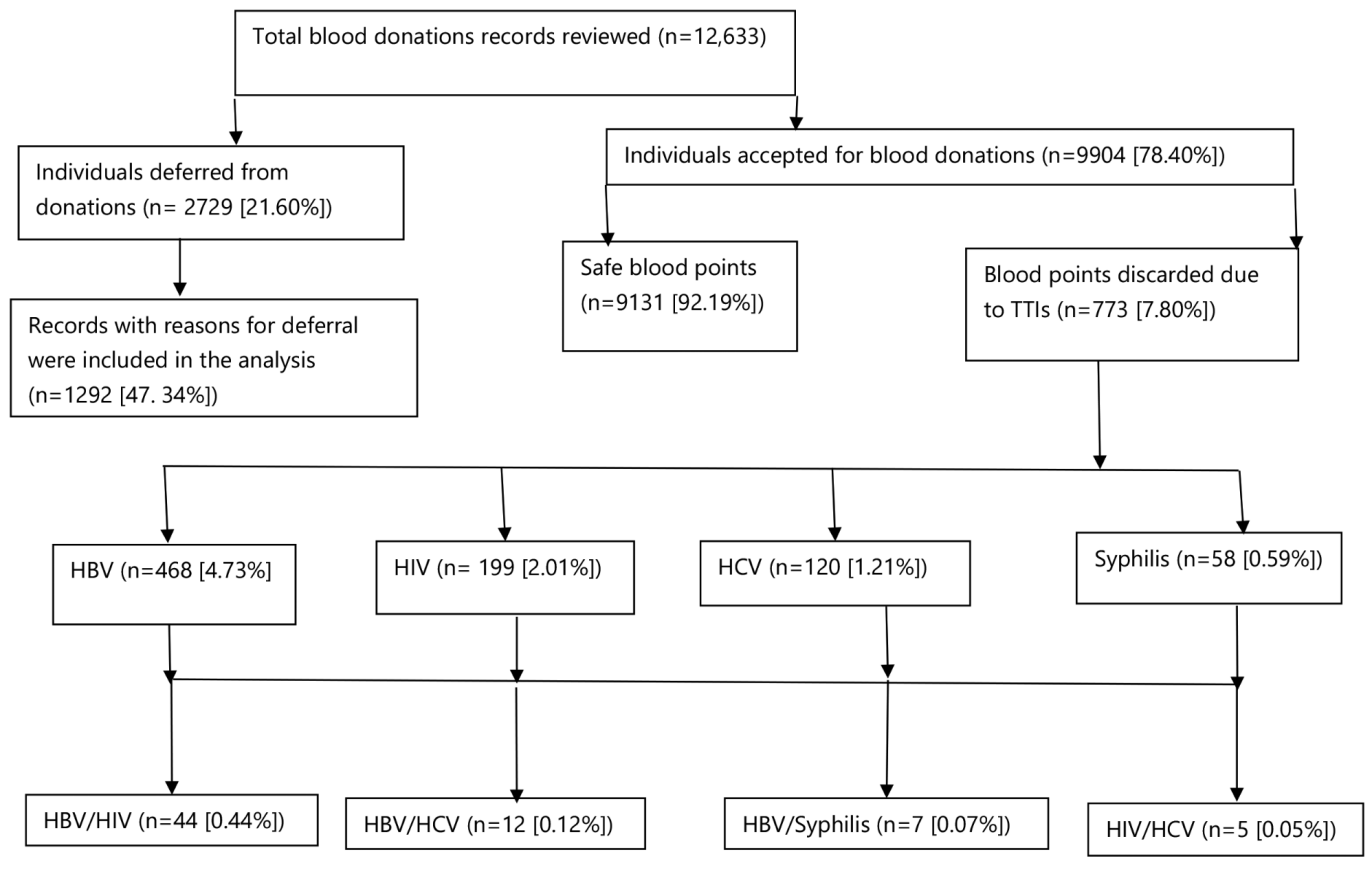
Summary of total blood records reviewed for pre-donation deferrals and transfusion transmissible infections (TTIs), Kwale satellite Blood Transfusion Center (KSBTC), Kenya, 2018–2022

A review was conducted on 12,633 blood donation records. Among these, individuals 2,729/12,633 (21.60%) were deferred from donating blood. Of those deferred, 1,292/2,729 (47.34%) were included in the final analysis because reasons for their pre-donation deferral were available. Among those who were accepted to donate blood, 773/9904 (7.80%) were unsafe blood points due to TTIs leading to the discarding of the blood (Fig. 2).

### Social demographic characteristics of the accepted donors

The median age of the blood donors was 24 (range 16–65 years), with the majority being single (63.63%). Male donors constituted the majority at 80.31%, voluntary donors accounted for 59.02%, and first-time donors made up 68.15% (Table 1).

**Table 1:** Distribution of transfusion transmitted infection by Socio-demographic characteristics at KSBTC, Kwale County, Kenya

Variables	Frequency	Percent (%)	HBV (*n* %)	HIV (*n* %)	HCV (*n* %)	Syphilis (*n* %)
**Sex**	*n* = 9853					
Male	7913	80.31	403 (5.09)	163 (2.06)	99 (1.25)	52 (0.66)
Female	1940	19.69	63 (3.25)	36 (1.86)	21 (1.08)	6 (0.31)
**Age**	*n* = 9934					
16–17	1098	11.21	150 (3.64)	6 (1.68)	11 (1.00)	5 (0.46)
18–25	4119	42.03	75 (5.66)	82 (1.99)	45 (1.09)	26 (0.63)
26–30	1326	13.53	121 (6.53)	34 (2.56)	21 (1.58)	3 (0.23)
31–40	1853	18.91	62 (5.93)	34 (1.83)	21 (1.13)	9 (0.49)
41–50	1046	10.67	24 (6.72)	24 (2.29)	15 (1.43)	8 (0.76)
> 50	357	3.64	33 (3.01)	17 (1.55)	6 (1.68)	5 (1.40)
**Marital status**	*n* = 9024					
Married	3282	36.37	203 (6.19)	74 (2.25)	34 (1.04)	19 (0.58)
Single	5742	63.63	217 (3.78)	108 (1.88)	68 (1.18)	30 (0.52)
**Level of education**	*n* = 9157					
None/Primary	1817	19.84	127 (6.99)	49 (2.70)	29 (1.60)	14 (0.77)
Secondary/Tertiary	7340	80.16	314 (4.28)	135 (1.84)	80 (1.09)	41 (0.56)
**Occupation**	*n* = 7505					
Non-students	3331	44.38	178 (5.34)	59 (1.77)	36 (1.08)	18 (0.54)
Students	4174	55.62	145 (3.47)	81 (1.94)	44 (1.05)	26 (0.62)
**Donation frequency**	*n* = 9259					
First timer	6310	68.15	299 (4.74)	142 (2.25)	74 (1.17)	37 (0.59)
Repeat	2949	31.85	140 (4.75)	44 (1.49)	27 (0.92)	17 (0.58)
**Donation strategy**	*n* = 9820					
Static	5430	55.3	250 (5.69)	96 (2.19)	70 (1.59)	23 (0.52)
Mobile	4390	44.7	209 (3.85)	102 (1.88)	48 (0.88)	35 (0.64)
**Type of donor**	*n* = 9670					
FRD	3963	40.98	232 (5.85)	88 (2.22)	64 (1.61)	21 (0.53)
Voluntary	5707	59.02	223 (3.91)	110 (1.93)	51 (0.89)	36 (0.63)

†† Any record that did not contain the specific part of the donor’s socio-demographic information was excluded from the analysis, therefore denominators differ from one variable to the next; FRD; family replacement donors, HBV; hepatitis b virus; HCV; hepatitis c virus; HIV; human immunodeficiency virus ††

The TTIs identified were due to cases of HBV, 468 (4.73%), HIV, 199 (2.01%), HCV, 120 (1.21%), and syphilis, 58 (0.59%) among donated blood units (Fig. 2).

The pre-donation deferral rate stood at 21.60%, with a median age of 20 (range 14 to 65 years). The primary reasons for deferral among prospective donors were low hemoglobin levels (51.86%), past medical history (13.47%), and high blood pressure (11.15%), among other conditions (Table 2).

**Table 2:** Reasons for pre-donation deferral, at KSBTC, Kwale County, Kenya, 2018–2022 (*n* = 1,284)

Serial No	Reason for Pre-donation Deferral	Frequency (*n*)	Percent (%)
1	Low hemoglobin	670	51.86
3	Past medical history	174	13.47
2	High blood pressure	144	11.15
6	High pulse rate	94	7.28
7	Age-related reasons	55	4.26
5	Blood group mismatch	29	2.24
4	Low blood pressure	25	1.93
8	Declined	25	1.93
11	Underweight	22	1.7
10	Physiological reasons	15	1.16
12	Invisible veins	9	0.7
9	Low pulse rate	8	0.62
14	History of use of hard drugs	7	0.54
15	History of recent vaccination	4	0.31
13	Short inter-donation interval	3	0.23

†† Note; the reasons for pre-donation deferrals may exceed the total number of prospective donors due to the reasons of deferrals were more than one for some prospective donors; Age-related reasons (under age and over age); physiological reasons (fasting, pregnancy, menses, breastfeeding)††

The bivariate analysis revealed that individuals with none or primary education were 1.7 times more likely to test positive for at least one TTI (COR = 1.7, 95% CI 1.39–1.96) compared to those with secondary or tertiary education. Male donors were 1.5 times more likely to test positive for at least one TTI (COR = 1.5, 95% CI 1.21–1.82) compared to female donors. Family or relative replacement donors were 1.5 times more likely to test positive for at least one TTI compared to voluntary donors. First-time blood donors had a 10% higher likelihood of testing positive for at least one TTI compared to repeat donors (COR = 1.1, 95% CI 0.96–1.34) (Table 3).

**Table 3:** Bivariate and multivariate potential risk factors associated with transmissible transfusion infections at KSBTC, 2018–2022 (*n* = 9904)

Variables	*N*	At least one TTI (*n* %)	cOR 95% CI	*p*-value	aOR 95% CI	*p*-value
**Sex**						
Male	7913	659 (8.33)	1.5 (1.21–1.82)	< 0.01	1.3 (1.01–1.57)	0.04
Female	1940	112 (5.77)	1		1	
**Age**						
16–17	1098	59 (5.37)	1			
18–25	4119	269 (6.53)	1.2 (0.92–1.64)	0.16	**	
26–30	1326	124 (9.35)	1.8 (1.32–2.50)	< 0.01	**	
31–40	1853	176 (9.50)	1.9 (1.36–2.51)	< 0.01	**	
41–50	1045	101 (9.67)	1.9 (1.35–2.63)	< 0.01	**	
> 50	357	37 (10.36)	2.0 (1.33–3.13)	< 0.01	**	
**Marital status**						
Married	3282	314 (9.57)	1.5 (1.29–1.77)	< 0.01	**	
Single	5742	375 (6.53	1			
**Level of education**						
None/Primary	1817	203 (11.17)	1.7 (1.39–1.96)	< 0.01	1.4 (1.11–1.68)	0.004
Secondary/Tertiary	7340	519 (7.07)	1		1	
**Occupation**						
Non-students	3331	277 (8.32)	1.4 (1.45–1.63)	< 0.01	**	
Students	4174	259 (6.21)	1			
**Donation frequency**						
First timer	6310	503 (7.97)	1.1 (0.96–1.34)	0.16	1.2 (1.01–1.44)	0.04
Repeat	2949	210 (7.12)	1		1	
**Donation Strategy**						
Static	4390	411 (9.36)	1.5 (1.29–1.74)	< 0.01	1.4 (1.12–1.63)	0.002
Mobile	5430	350 (6.45)	1		1	
**Type of donor**						
FRD	3963	380 (9.59)	1.5 (1.30–1.76)	< 0.01	**	
Voluntary	5707	374 (6.55)	1			

††CI; confidence interval, cOR; crude odds ratio; aOR; adjusted odds ratio; N; total observations; n; sample that had TTI; TTI; transmissible transfusion infections; FRD; family replacement donors††

Male donors (aOR = 1.3, 95% CI 1.01–1.57), donors with none or primary education level (aOR = 1.4 95% CI 1.11–1.68), first timer donors (aOR = 1.2, 95% CI 1.01–1.44), and static strategy for blood collection (aOR = 1.4, 95%CI 1.12–1.63) were independently associated with testing positive for at least one TTI (Table 3).

## Discussion

This study documented a pre-donation deferral rate of 21.60% which was within the WHO range of less than 1% to over 37% [13]. However, the rate was higher compared to other studies done in various transfusion programs where the rate ranged from 4 to 16% [1–5]. Studies in Asia also reported a lower pre-donation deferral rate of between 4.3% and 19.2% [17–19]. The differences in the deferral rates may be due to the differences in donor selection criteria or the donor population used in the different transfusion programs. The high deferral rate review of the donor deferral process to minimize psychological effects or other effects that may prevent donors from returning for future donations.

Low haemoglobin was the main cause of the pre-donation deferrals at 51.86%. In contrast a lower pre-donation deferral rate due to low haemoglobin has been reported in Asia between 4.3% and 19.2% [17–19]. Given the epidemiological context in the study area, the low hemoglobin could be because of parasitic infections including malaria, soil-transmitted helminths, and poor nutrition status [20, 21], where these conditions are endemic in the study settings. The implication of this finding is a high prevalence of anemia in the studied population.

Also, Hypertension was reported as a common cause of deferral at 11.15%. This finding is in agreement with other studies, which reported proportions of 12.3–14.6% [17, 19, 22]. Indeed, different rates of hypertension have been reported in different populations of prospective blood donors around the globe ranging from 5.8 to 29.4% [1–5, 17, 19, 22].

The majority of the pre-donation deferrals were for short-term temporary deferrals such as anemia, underweight, recent vaccination, underage, physiological reasons, difficulty in locating veins, and Short inter-donation intervals that can be resolved in days or months, after which the donors can return to donate [1].

Post-donation deferral was mainly due to TTIs. The KBSTC like most blood transfusion programs screens for TTIs which have a residue risk of transmitting blood-borne infections such as HBV, HCV, HIV, and syphilis during transfusions. These infections pose significant challenges in ensuring the safety and availability of the blood supply for recipients. The rate of blood donors testing positive for at least one TTI was 7.80%, consistent with the World Health Organization’s estimated TTI prevalence in Kenya, which is 5.3% []. Additionally, other blood transfusion facilities in Kenya have recorded comparable percentages of TTIs among blood donors, varying between 9.4% and 14.1% [6, 9, 23]. In sub-Saharan Africa, various countries have documented similar ranges of TTI positivity rates among blood donors, ranging from 3.7 to 8.8% [8, 24, 25].

In our study, the occurrence of HBV infections was 4.73% among blood donors. A study conducted in the same study site found a higher proportion of HBV infections at 8.8% among voluntary blood donors who were school-going adolescents [10]. In other parts of Kenya, where investigations among blood donors have been conducted, the rates were similar, ranging from 2.3 to 5.6% [6–10]. From an epidemiological viewpoint, both our findings and other studies conducted in Kenya suggest an intermediate level of endemicity for HBV infections, with a seroprevalence range of 2–7% [26]. Despite this, the reported rate of HBV infections among blood donors in our study is lower compared to findings in other African studies, where high endemicity of HBV seroprevalence, exceeding or equal to 8%, was reported with rates ranging from 11.8 to 13.4% [6–10].

The HIV infection rate was 2.01% which was comparable to studies carried out in Kenya where a positivity rate ranging from 0.9 to 5% for blood donors with HIV infections in various transfusing facilities was reported [6–10].

The rate for HCV was 1.21% among blood donors, which aligns with findings from other cross-sectional studies conducted at different regional blood donation sites in Kenya with a positivity rate for HCV infections ranging from 0.5 to 3.2% [6–10].

The overall prevalence of Syphilis infections among our blood donor population stood at 0.59%, similarly, Syphilis infections within the blood donor population in Kenya had been reported ranging from 0.6 to 3% [6–10]. All these statistics are consistent with WHO reports which reported in lower-middle-income countries like Kenya, HBV infections among blood donors range from 0.70 to 4.74%, HIV infections range from 0.04 to 0.62%, HCV infections range from 0.12 to 0.99%, and Syphilis infections range from 0.19 to 1.38% [15].

The differences in seroprevalence rates can be attributed to variations in geographical locations, the endemicity of blood-borne pathogens in specific populations, and the sensitivity of the screening techniques used to detect serological markers in the respective studies.

This study indicated that a greater percentage of male donors (8.33%) had at least one TTI compared to their female counterparts (5.77%). Male donors exhibited a 30% higher likelihood of having at least one TTI compared to female donors, which was statistically significant *(p-value 0.04).* The comparison of TTI to sex-related analysis may be interpreted cautiously due to the higher dominance of male donors (80.31%) in the current study thus it may confound the interpretation. Nevertheless, a study in Ethiopia reported the positivity rates of TTIs were significantly higher among male than female blood donors (*p*-value = 0.021) [27]. Similarly, In Eretria, a study reported male donors had a significantly higher frequency of TTIs compared to female donors (4.0% versus 2.97%, *p* < 0.00) [12]. In contrast in Kenya, the study reported no associations between the frequency of TTIs for male (1.9%) versus female (1.5%) donors *p* = 0.470 [8]. Additionally, another study found that female voluntary donors had a 40% higher likelihood of testing positive for HBV infections compared to male donors (*p* = 0.29), but this difference did not reach statistical significant [10].

The current study reports that 80.16% of the blood donors had secondary or tertiary education. In the Kenyan context, blood is primarily obtained from adolescents, particularly those in educational institutions [28]. This circumstance could elucidate the elevated literacy rate observed in the study population, with a notable 55.62% of pool donors identified as students.

Regarding donors testing positive for at least one TTI, the study shows that donors with none or primary education and lower were 40% likely to test positive for at least one TTI compared to those with secondary or tertiary education which was statistically significant *(p value = 0.004*). In line with our study, a study in Ethiopia reported those blood donors with no or low level of education (AOR = 16.95, 95% CI = 1.66–172.9, *p* = 0.017) were at higher risk of HBV infection compared to literate blood donors [29]. In contrast to our study, a study in India reported no statistically significant correlation was seen between the education status of donors and TTIs [30]. In Kenya, blood donors with no or only primary education (aOR = 9.05; *p*-value = 0.0262) were found to have a higher association with HIV infections compared to those with secondary or tertiary education [6]. The study in Iran revealed that a lower educational level was significantly associated with higher rates of HBV (*p* < 0.001, OR: 2.14; CI = 1.76–2.6) and HCV (*p* = 0.02, OR: 2.21; CI = 1.13–4.31) infections compared to donors with higher education [31]. The odds of having one TTI among blood donors with no formal education were 4.84 times (AOR = 4.84; 95% CI: 1.09–21.46; *p* < 0.038) higher than those of persons who learned secondary and above [32].

First-time donors had 20% higher odds of testing positive for at least one TTI, in comparison to repeat donors. In contrast to our study, in Tanzania, a study found that the prevalence of TTIs was higher among repeat donors (7.9%, 95% CI: 7.0-8.9) compared to first-time donors (8.9%, 95% CI: 8.0–10.0) [33]. However, HBV infections were more common among first-time blood donors than repeat donors, with rates of 4.7% versus 3.4%, respectively (adjusted *p* = 0.003) [33]. In Kenya, the study reported no associations between the frequency of TTIs for first-time (3.1%) versus repeat (03%) donors *p* = 0.156 [8], while another study revealed that first-time blood donors had a 50% higher chance of testing positive for HBV infections compared to repeat donors (cPOR = 1.5, 95% CI 0.61–3.57, *p*-value 0.38) but were not statistically significant [10]. Similarly to our study, several studies in Kenya reported that first-time donors were more likely to return a positive test for TTIs than repeat donors [9, 10, 28].

Concerning donor types, our study reported FRDs had a 50% higher likelihood of having at least one TTI compared to VNRDs. In contrast a study conducted in Angola found no statistically significant difference in the prevalence of TTI markers between FRDs and VNRDs: (50.4% vs. 40.9%, *p* < 0.126 for Hepatitis B); for Hepatitis C (5.2% vs. 3.0, *p* < 0.774); for HIV (7.1% vs. 3.0% *p* < 0.323) and Syphilis 20% vs. 19.2%, *p* < 0.894 [34]. In line with our study, the study conducted in Eritrea found that FRDs had a higher frequency of TTIs compared to VNRDs (6.9% versus 3.4%, *p* < 0.000). Specifically, the prevalence of TTI markers was significantly higher in FRDs compared to VNRDs for HBV (3.6% versus 1.9%, *p* < 0.000), HCV (1.0% versus 0.7%, *p* < 0.006), HIV (0.6% versus 0.3%, *p* < 0.000), and another HIV marker (1.7% versus 0.5%, *p* < 0.000) [12]. In Tanzania, a study reported that the likelihood of TTIs in FRDs was more than double compared to VNRDs, with rates of 9.0% (95% CI: 8.3–9.8) in FRDs and 4.1% (95% CI: 2.8–5.7) in VNRDs (*P* < 0.001) [33].

The current study serves to shed some light on the characteristics of potentially high-risk blood donor populations, by quantifying the causes for both pre and post-donation deferrals. This method provides the basis for developing a crucial tool for advancing blood safety and contributing to policy development.

The current study employed a census-type sampling technique with a substantially large sample size, in this way; potential selection bias was minimized while ensuring the inclusion of well-defined study population thus enhancing comparability. Indeed, these findings serve as valuable updates in the area of blood donation and transfusion practice and contribute essential information for the KSBTC, the Department of Health in Kwale County, and the KTTA under the Ministry of Health.

Several limitations are however noted; the retrospective nature of our study and data limits the variables that could have been analyzed. Moreover in the dataset, for pre-donation deferral, slightly above half of the cases had no reasons for deferral and could therefore not be included in the analysis. Also, there is an inherent drawback in prevalence studies when determining causality, in this case between TTIs and the associated risk factors. The seroprevalence identified through the Elisa method employed in this study may not necessarily indicate an ongoing or current infection. The interpretation of these results is confined to the local context, potentially restricting their applicability to the broader community. Nevertheless, the assessment of the prevalence and factors associated with TTIs within the specific satellite blood bank center indeed serves as a reliable indicator within the context of the surrounding community and offers valuable insights into the broader community factors for donor deferral.

## Conclusion

This study reveals that a significant portion of the voluntary blood donors were young individuals, predominantly students, with males comprising the majority of donors. The rate of pre-donation deferrals was high; the causes included low HB, past medical history, and hypertension among others. The TTIs were the main cause of post-donation deferrals with HBV being the predominant. Male donors, individuals with no formal education or only primary education, first-time donors, and the use of a static strategy for blood collection were identified as potential risk factors independently linked with TTIs.

There is a need for a broad public education campaign regarding the value, simplicity, and low danger of blood donation. The policymakers need to routinely review the criteria and devise strategies to ensure follow-up of the temporary deferrals to mitigate the loss of potential donors. This approach will also contribute to advancing our comprehension of the criteria for selecting blood donors in the study region for the future.

### Electronic supplementary material

Below is the link to the electronic supplementary material.

Supplementary Material 1

Supplementary Material 2

Supplementary Material 3

## Data Availability

The datasets used and/or analyzed during the current study are available from the corresponding author on reasonable request. (Data sets are provided within the supplementary information files).

## Abbreviations

AOR: Adjusted Odds Ratio
CI: confidence intervals
COR: Crude Odds Ratio
FRDs: Family/Relative replacement donors
HBV: Hepatitis B Virus
HCV: Hepatitis C Virus
KNSBTS: Kenya National Standards for Blood Transfusion Services
KSBTC: Kwale Satellite Blood Transfusion Center
KTTA: Kenya Tissue and Transplant Authority
MCRH: Msambweni County Referral Hospital
SD: Standard Deviations
TTIs: Transfusion-Transmissible Infections
VNRDs: Voluntary non-remunerated blood donors
