# Supplementary material for: Causes of pre and post-donation deferrals among blood donors, at Kwale Satellite Blood Transfusion Center, Kwale County, Kenya, 2018–2022
Source: BMC Public Health. 2024 Aug 13;24:2197. doi: 10.1186/s12889-024-19535-1 (PMC11321081; doi:10.1186/s12889-024-19535-1)
Supplement: Supplementary file 3 — Supplementary Material 3 [file 12889_2024_19535_MOESM3_ESM.pdf]

Kenya Tissue and Transplant Authority

DamuKE
The Kenya Blood Banking Management System

REPUBLIC OF KENYA

KENYA TISSUE AND TRANSPLANT AUTHORITY (KTTA)

BLOOD DONOR QUESTIONNAIRE

Name of Blood Establishment:	**KWALE SATELLITE**	Affix/write Donation Number here
Blood Establishment Code:	**005**	

Donation site: ______________________________ **County:** _________________ **Donor Number:** ______________

SECTION 1: DONOR INFORMATION (To be completed by the donor)

Surname	Other Names	Gender (F/M/Other/Prefer not to disclose)

Nex of kin name: _____________________ **Cell No.** _______________ **R/ship**____________ **Residence**__________

Unique Identifier (Student No. /ID No./PP No.)**:** ________________ **Date of Birth:** ______ DD / MM / YY

Status: (Mark in appropriate box): Single/Married /Separated /Divorced /Widowed/Widower /Prefer not to disclose

Cell Phone No.	Email	County	Sub-county	Ward

Level of Education: (Mark in appropriate box): None/ Primary/ Secondary/ Tertiary/ other

Occupation: (Mark in appropriate box): Employed/ Self-Employed/ Unemployed/ Student

When did you last donate blood?	Number of previous donations:_______
☐ Never donated ☐ 3 months ☐ 6 months ☐ 12 months ☐ More than 12 months	

SECTION 2: ELIGIBILITY QUESTIONNAIRE (Please fill in truthfully and accurately- your answers will be treated with utmost confidentiality- tick where appropriate)

1	Are you feeling well today?	Yes / No
2	Have you eaten in the last 6 hours?	Yes / No
3	Have you ever fainted within the past one year?	Yes / No
4	Are you pregnant or lactating	Yes / No
5	Have you traveled in the last 14 days outside your usual area of residence?	Yes / No
6	Are you on any regular medication, antibiotics, analgesics e.g., Aspirin, any other?	Yes / No
7	In the last 3 months have you had any tattooing or body piercing e.g., ear piercing?	Yes / No
8	In the last 3 months have you had sexual activity with a person whose health status you do not know?	Yes / No
9	In the last 3 months have you had a vaccination	Yes / No
**In the past 6 months have you:**		
10	Had surgery or medical treatment	Yes / No
11	Received Blood or Blood Products	Yes / No
**Have you ever:**		
12	Had Hepatitis or yellow eyes?	Yes / No
13	Been exposed to suspected case of Covid-19 in the last 14 days?	Yes / No

SECTION 3: DECLARATION & CONSENT (Please read this before you sign the form)

i.	I declare that I have filled in all the information required in this form truthfully and accurately.
ii.	I declare that I will also answer truthfully and accurately to any other questions that may be asked of me, for purposes of verifying my eligibility to donate blood.
iii.	I consent to give blood; I understand that if found to be safe, it may be used for transfusion for the benefit of others.
iv.	The undersigned hereby releases the Kenya Tissue and Transplant Authority, its agents or employees, as well as any other users and exhibitors of said pictures, from any and all claims, demands, accountings and causes for which the aforesaid videotape, testimonial, motion picture, digital images, or photograph likeness may be used pursuant to this consent and general release. It is also my understanding that i will receive no compensation for my likeness and testimonial.
v.	I understand that whatever blood I donate will be screened for HIV, Hepatitis B & C, and Syphilis and the results of my tests may be obtained from the Kenya Tissue and Transplant Authority by myself.
vi.	I understand that should any of the screening tests give a reactive result, I will be contacted by use of any communication medium(s), to send **me important information and or to offer me counselling to make an informed decision** about further confirmatory testing and management. Such medium(s) shall include but are not limited to e-mail, post office, mobile telephone and/or fixed telephone.
vii.	I hereby give consent to KTTA to use the contact details provided in this form to communicate to me as the need may be.
viii.	I understand the blood may be used for scientific research, main objective being to improve the safety of the blood supply to patients. I further understand that this will be done in an anonymized manner, so as to not to reveal Personal Identifiable Information (PII), that may be linked directly to me No☐ Yes ☐

Donor Signature:____________________Date:____________________

SECTION 4: FOR OFFICIAL USE

a. Donor Screening Report

Weight (kg)	Hb >12.5g/dl	BP	Pulse	Temp (°C)	Blood Group	Donor is Eligible	
						Yes	No

Donor Deferred (Y/N)	Brief reason for deferral

Type of donor (Replacement/ Voluntary/ Autologous)	Type of Donation (Normal / Apheresis)

Name of Nurse / Counselor:____________________Date:____________Sig_________________

b. Donation outcome & post donation adverse events: (Yes or no)

Underweight Unit (Y/N)	Underweight Unit (Y/N)	> 1Venipuncture (Y/N)	Hematoma (Y/N)	Fainting (Y/N)	Nausea (Y/N)

Vomiting (Y/N)	Headache (Y/N)	Convulsion (Y/N)	Incontinence of urine/stool (Y/N)

Time Needle In		Time Needle Out		Volume donated (ml)		Bag Type	

SECTION 5: NOTIFICATION

Reported by:______________________Date:___________________Sign:________________________
